# Supplementary material for: HCNetlas: A reference database of human cell type-specific gene networks to aid disease genetic analyses
Source: PLoS Biol. 2025 Feb 5;23(2):e3002702. doi: 10.1371/journal.pbio.3002702 (PMC11798474; doi:10.1371/journal.pbio.3002702)
Supplement: S1 Fig — (A) Scatter plot depicting the relationship between the number of cells used for network inference (cell count) and network size by node or edge count of CGNs. (B) Uniform Manifold Approximation and Projection (UMAP) visualization of T cell CGNs based on the network node profiles, where each circle’s size represents the number of network genes and different colors correspond to various T cell subtypes. (C) UMAP plot displaying T cell CGNs across different organs and tissues. The shapes of the points distinguish between different organs, while the color denotes the specific tissue types. The size of each point corresponds to the size of CGN, as determined by the number of genes. The data underlying this figure can be found in https://doi.org/10.5281/zenodo.14522296. (PDF) [file pbio.3002702.s001.pdf]

Supplementary Figures

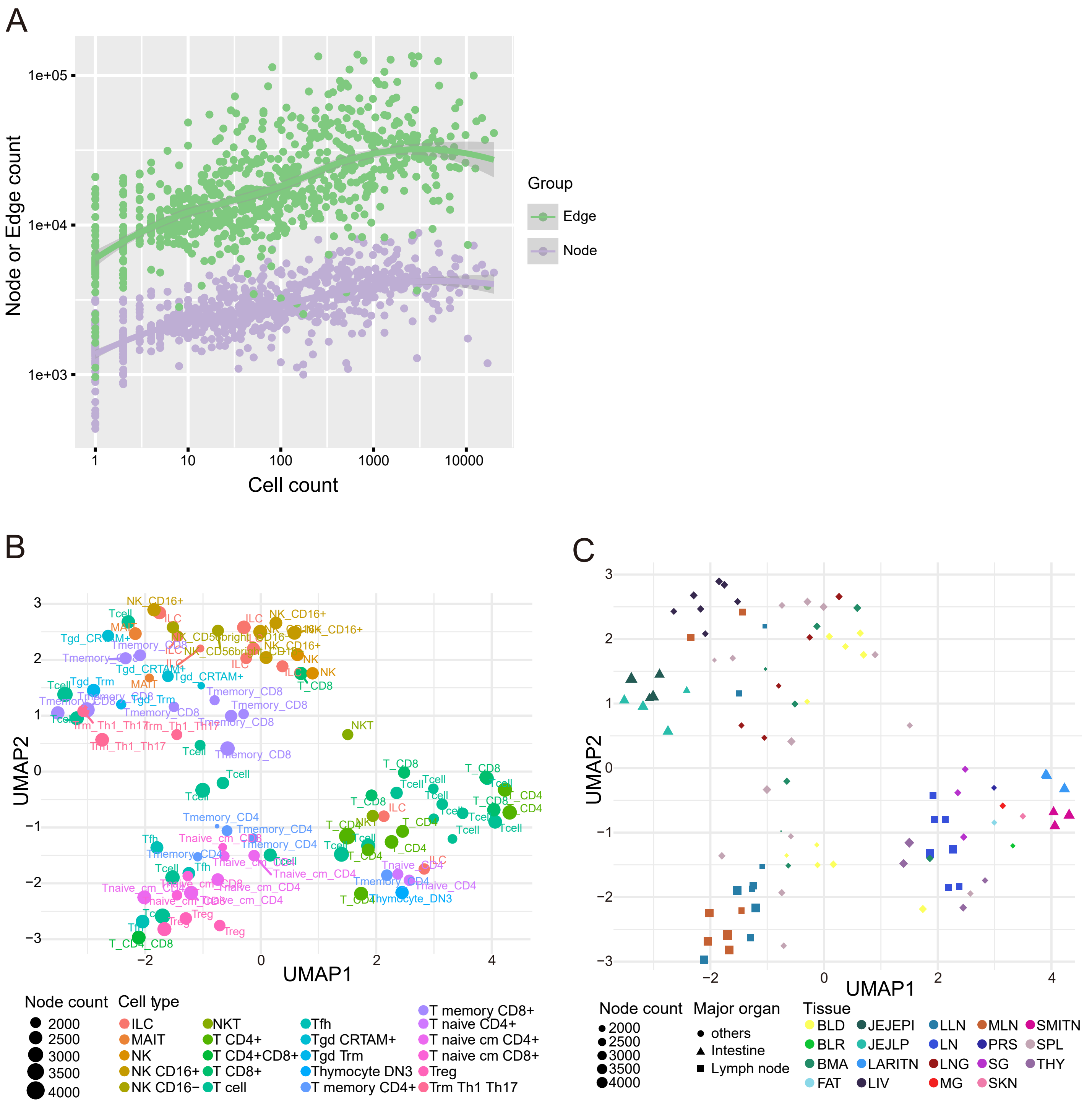

Figure S1. Overview of cell-type-specific networks (CGNs) of the HCNetlas.

A. Scatter plot depicting the relationship between the number of cells used for network inference (cell count) and network size by node or edge count of CGNs. **B**. Uniform Manifold Approximation and Projection (UMAP) visualization of T cell CGNs based on the network node
