# Supplementary figures and images for: HCNetlas: A reference database of human cell type-specific gene networks to aid disease genetic analyses
Source: PLoS Biol. 2025 Feb 5;23(2):e3002702. doi: 10.1371/journal.pbio.3002702 (PMC11798474; doi:10.1371/journal.pbio.3002702)

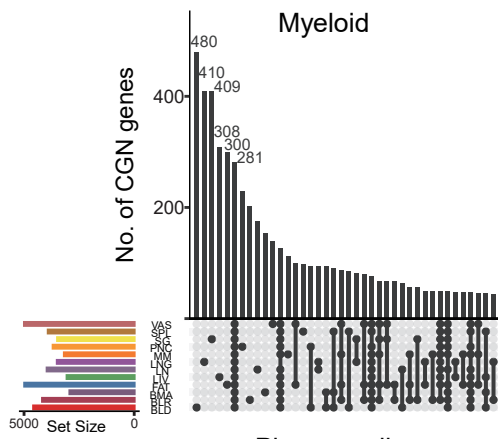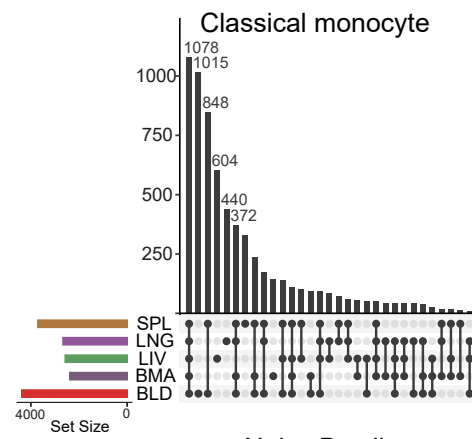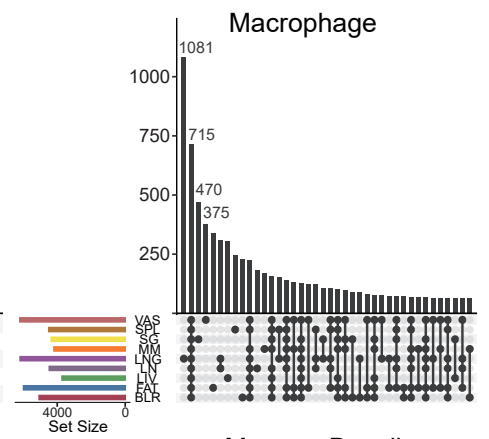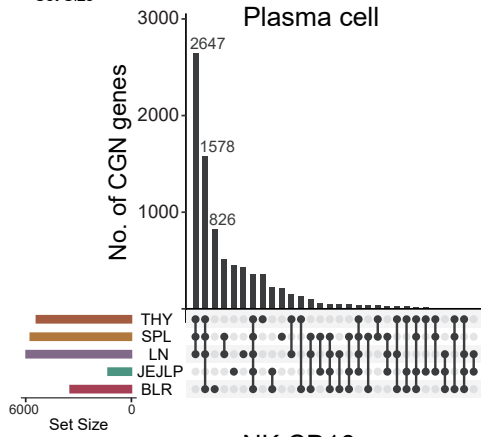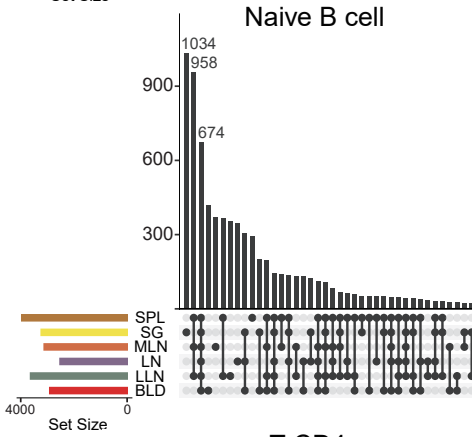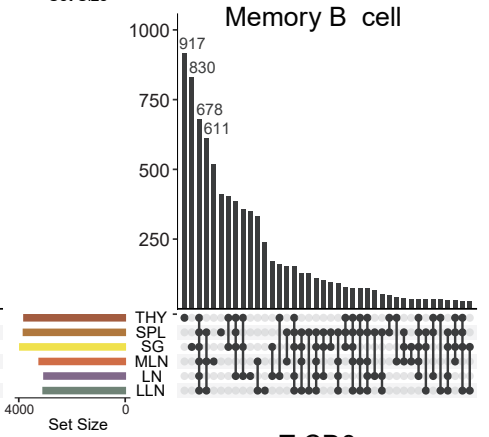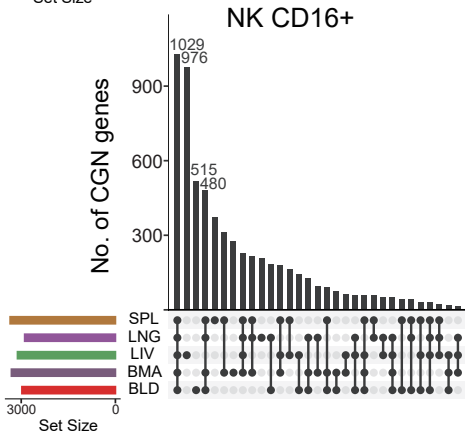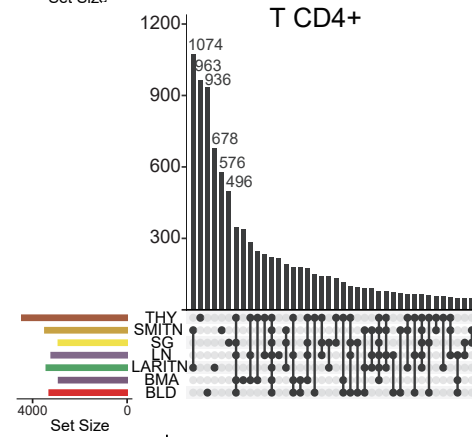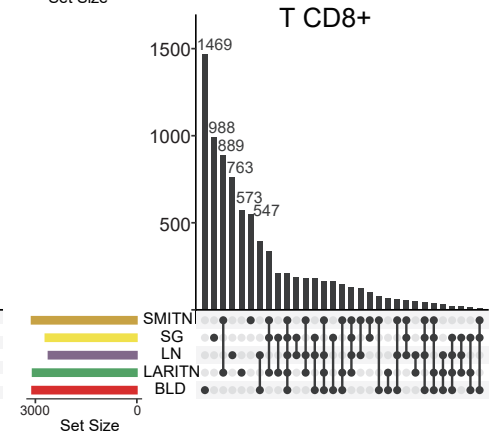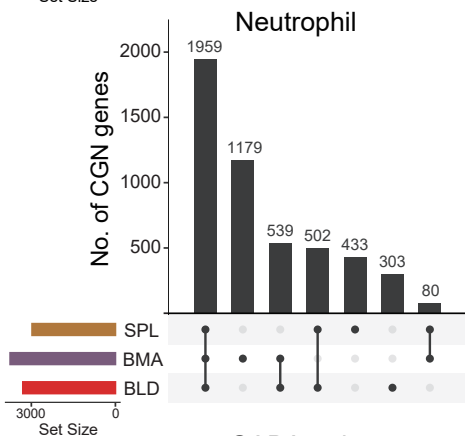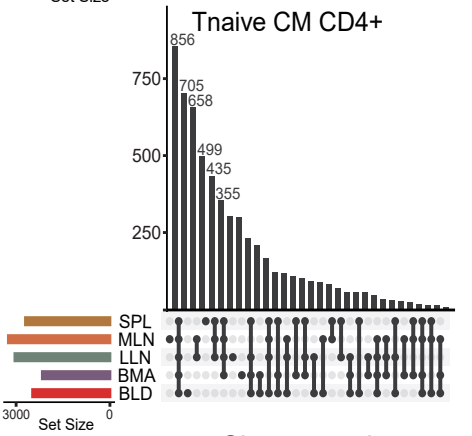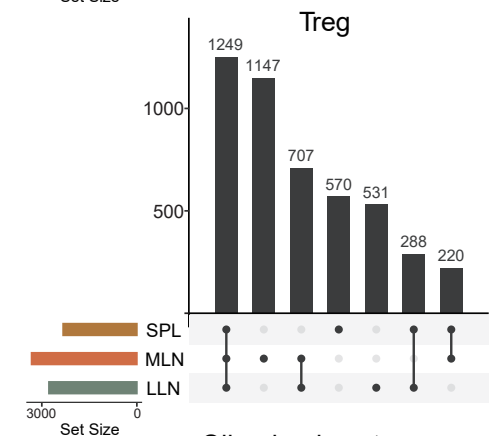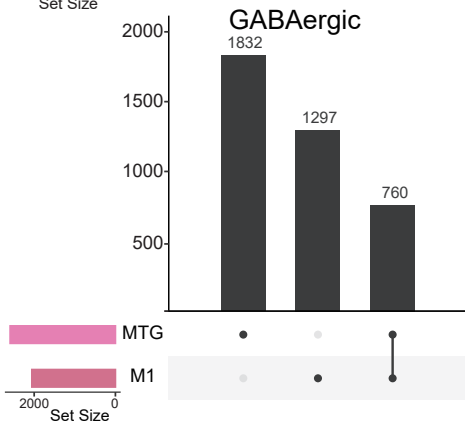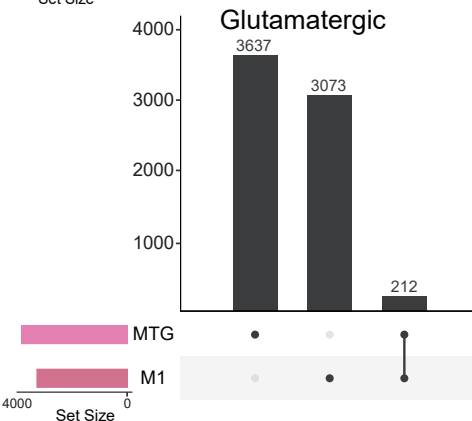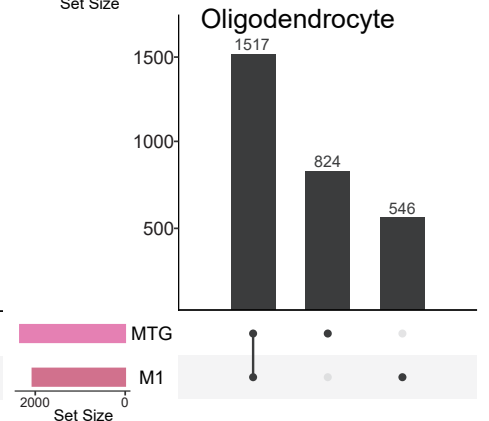

Supplement: S3 Fig — Upset plots illustrate the intersection of network genes across CGNs for various tissues within each cell type. The data underlying this figure can be found in https://doi.org/10.5281/zenodo.14522296. (PDF) [file pbio.3002702.s003.pdf]

A

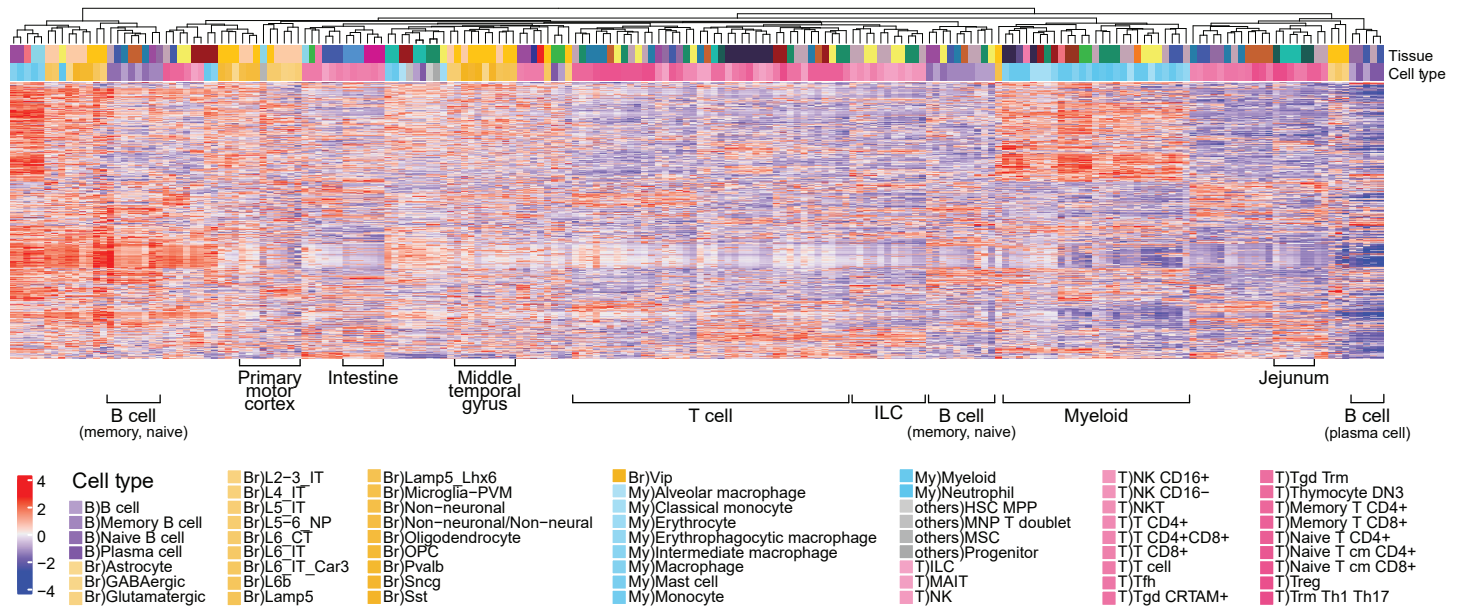

B

## Schizophrenia (GWASCAT DIS 674)

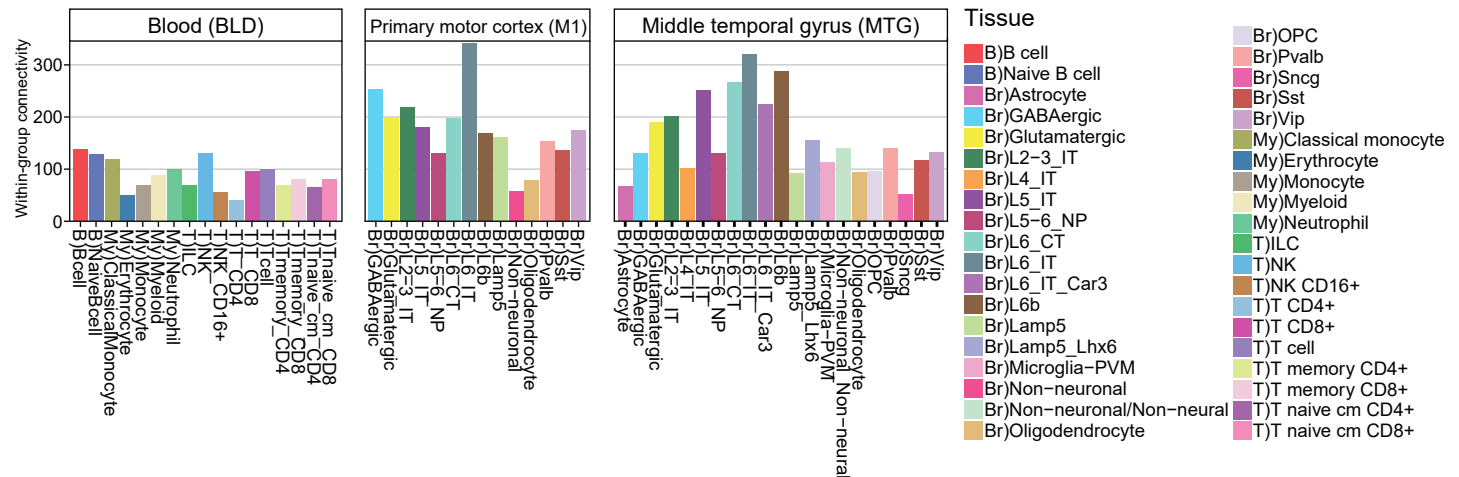

C

## AD associated genes (GWASCAT 50 terms)

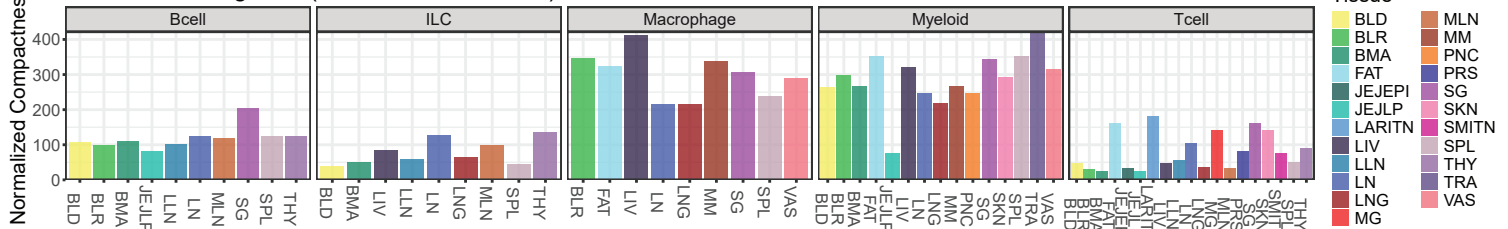

Supplement: S4 Fig — (A) Heatmap displaying the disease profiles of various cell types across different tissues, conducted with gene set variation analysis (GSVA). Each column represents a CGN of HCNetlas, while each row corresponds to a disease gene set sourced from either DisGeNET or GWAS Catalog. Color intensity indicates the degree of association of the CGN signature genes with each disease gene set. (B, C) Bar graphs showing the within-group connectivity of genes associated with Schizophrenia (B) and Alzheimer’s Disease (C) across different cell lineages or tissues. These disease-associated genes were collected from GWAS catalog. The data underlying this figure can be found in https://doi.org/10.5281/zenodo.14522296. (PDF) [file pbio.3002702.s004.pdf]
