# Supplementary material for: HCNetlas: A reference database of human cell type-specific gene networks to aid disease genetic analyses
Source: PLoS Biol. 2025 Feb 5;23(2):e3002702. doi: 10.1371/journal.pbio.3002702 (PMC11798474; doi:10.1371/journal.pbio.3002702)
Supplement: S5 Fig — Evaluation of HCNetlas for cell-type resolved disease genetics for Alzheimer’s disease (AD). (A) Venn diagram displaying overlap between AD-associated genes predicted by differential hubness and differential expression. (B) Networks of signature genes by top 10 hub genes for reference cell type-specific gene network (CGN) from HCNetlas and disease CGNs from disease samples for inhibitory neuron or excitatory neuron. The data underlying this figure can be found in https://doi.org/10.5281/zenodo.14522296. (PDF) [file pbio.3002702.s005.pdf]

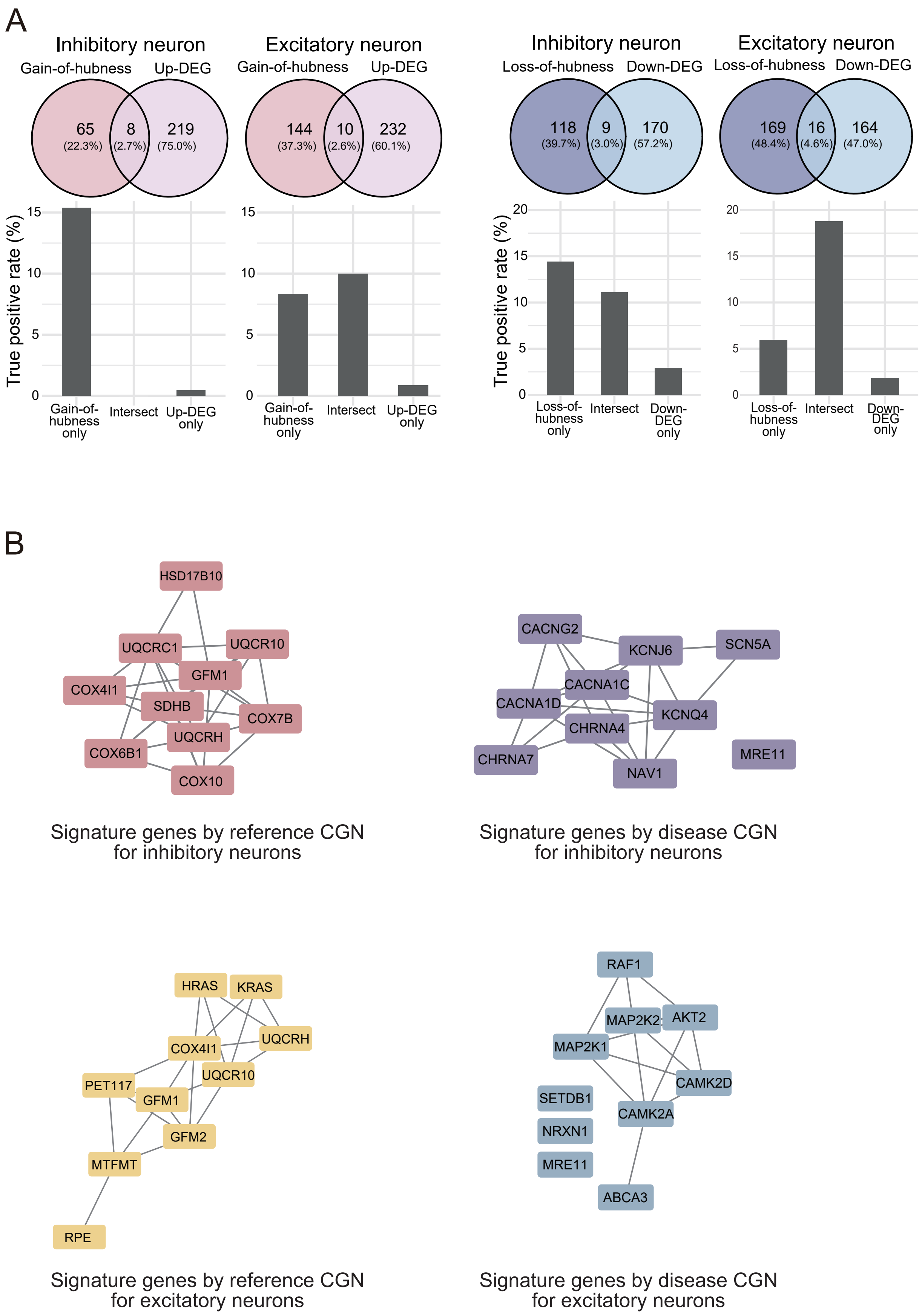

A
Inhibitory neuron
Gain-of-hubness
Up-DEG
65 (22.3%)
8 (2.7%)
219 (75.0%)
Excitatory neuron
Gain-of-hubness
Up-DEG
144 (37.3%)
10 (2.6%)
232 (60.1%)
Inhibitory neuron
Loss-of-hubness
Down-DEG
118 (39.7%)
9 (3.0%)
170 (57.2%)
Excitatory neuron
Loss-of-hubness
Down-DEG
169 (48.4%)
16 (4.6%)
164 (47.0%)
True positive rate (%)
0
5
10
15
20
Gain-of-hubness only
Intersect
Up-DEG only
Loss-of-hubness only
Down-DEG only
B
HSD17B10
UQCRC1
UQCR10
GFM1
COX4I1
SDHB
COX7B
UQCRH
COX6B1
COX10
Signature genes by reference CGN for inhibitory neurons
CACNG2
KCNJ6
SCN5A
CACNA1C
CACNA1D
KCNQ4
CHRNA4
CHRNA7
NAV1
MRE11
Signature genes by disease CGN for inhibitory neurons
HRAS
KRAS
COX4I1
UQCRH
UQCR10
PET117
GFM1
GFM2
MTFMT
RPE
Signature genes by reference CGN for excitatory neurons
RAF1
MAP2K2
AKT2
MAP2K1
CAMK2D
SETDB1
CAMK2A
NRXN1
MRE11
ABCA3
Signature genes by disease CGN for excitatory neurons
