# Supplementary material for: HCNetlas: A reference database of human cell type-specific gene networks to aid disease genetic analyses
Source: PLoS Biol. 2025 Feb 5;23(2):e3002702. doi: 10.1371/journal.pbio.3002702 (PMC11798474; doi:10.1371/journal.pbio.3002702)
Supplement: S2 Fig — (A) Box plot depicting the interconnectivity distribution of B cell and T cell marker genes within CGNs of the HCNetlas. Marker genes for each cell type were derived from gene sets in the Azimuth and CellMarker databases. (B) Heat map displaying the relative percentile ranks of the top 15 hub genes across multiple cell types and tissues, with values scaled per row and color intensity indicating the expression level from low (blue) to high (red). The data underlying this figure can be found in https://doi.org/10.5281/zenodo.14522296. (PDF) [file pbio.3002702.s002.pdf]

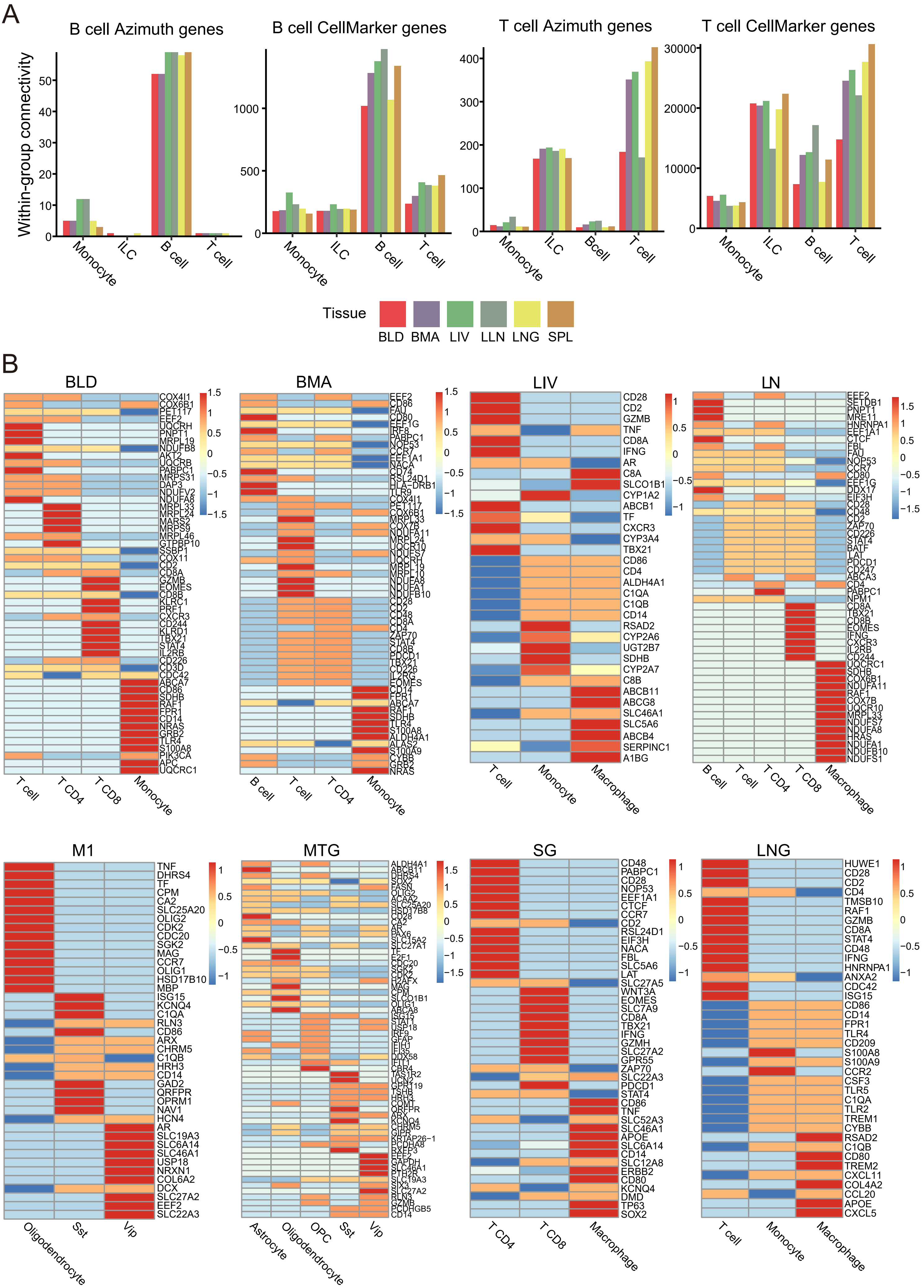
A
B cell Azimuth genes
B cell CellMarker genes
T cell Azimuth genes
T cell CellMarker genes
Within-group connectivity
0
10
20
30
40
50
500
1000
100
200
300
400
10000
20000
30000
Monocyte
ILC
B cell
Bcell
T cell
Tissue
BLD
BMA
LIV
LLN
LNG
SPL
B
BLD
COX4I1
COX6B1
PET117
EEF2
UQCRH
PNPT1
MRPL19
NDUFB8
AKT2
UQCRB
PABPC1
DAP3
NDUFV2
NDUFA8
MRPL33
MRPL24
MARS2
MRPS9
MRPL46
GTPBP10
SSBP1
COX11
CD2
CD8A
GZMB
EOMES
CD8B
KLRC1
PRF1
CXCR3
CD244
KLRD1
TBX21
STAT4
IL2RB
CD226
CD3D
CDC42
ABCA7
CD86
SDHB
RAF1
FPR1
CD14
NRAS
GRB2
TLR4
S100A8
PIK3CA
APC
UQCRC1
1.5
1
0.5
0
−0.5
−1
−1.5
T cell
T CD4
T CD8
Monocyte
BMA
EEF2
CD86
FAU
CD80
EEF1G
IRF8
PABPC1
NOP53
CCR7
EEF1A1
NACA
CD74
RSL24D1
HLA−DRB1
TLR9
COX4I1
PET117
COX6B1
MRPL33
COX7B
NDUFA11
MRPL24
UQCR10
NDUFS7
UQCRH
MRPL19
MRPL10
NDUFA8
NDUFA1
NDUFB10
CD28
CD2
CD48
CD8A
CD4
ZAP70
STAT4
CD8B
PDCD1
TBX21
CD226
IL2RG
EOMES
CD14
FPR1
ABCA7
RAF1
SDHB
TLR4
S100A8
ALDH4A1
ALAS2
S100A9
CYBB
GRB2
NRAS
B cell
T cell
T CD4
Monocyte
LIV
CD28
CD2
GZMB
TNF
CD8A
IFNG
AR
C8A
SLCO1B1
CYP1A2
ABCB1
TF
CXCR3
CYP3A4
TBX21
CD86
CD4
ALDH4A1
C1QA
C1QB
CD14
RSAD2
CYP2A6
UGT2B7
SDHB
CYP2A7
C8B
ABCB11
ABCG8
SLC46A1
SLC5A6
ABCB4
SERPINC1
A1BG
1
0.5
0
−0.5
−1
T cell
Monocyte
Macrophage
LN
EEF2
SETDB1
PNPT1
MRE11
HNRNPA1
EEF1A1
CTCF
FBL
FAU
NOP53
CCR7
CD80
EEF1G
DDX17
EIF3H
CD28
CD48
CD2
ZAP70
CD226
STAT4
BATF
LAT
PDCD1
CD247
ABCA3
CD4
PABPC1
NPM1
CD8A
TBX21
CD8B
EOMES
IFNG
CXCR3
IL2RB
CD244
UQCRC1
SDHB
COX6B1
NDUFA11
RAF1
COX7B
UQCR10
MRPL33
NDUFS7
NDUFA8
HRAS
NDUFA1
NDUFB10
NDUFS1
B cell
T cell
T CD4
T CD8
Macrophage
M1
TNF
DHRS4
TF
CPM
CA2
SLC25A20
OLIG2
CDK2
CDC20
SGK2
MAG
CCR7
OLIG1
HSD17B10
MBP
ISG15
KCNQ4
C1QA
RLN3
CD86
ARX
CHRM5
C1QB
HRH3
CD14
GAD2
QRFPR
OPRM1
NAV1
HCN4
AR
SLC19A3
SLC6A14
SLC46A1
USP18
NRXN1
COL6A2
DCX
SLC27A2
EEF2
SLC22A3
Oligodendrocyte
Sst
Vip
MTG
ALDH4A1
ABCB11
DHRS4
SOX2
FASN
OLIG2
ACAA2
SLC25A20
HSD17B8
CD28
CA2
AR
PAX6
SLC15A2
SLC27A1
TF
E2F1
CDC20
SGK2
CDK2
H2AFX
MAG
CPM
SLCO1B1
OLIG1
ABCA8
ISG15
STAT1
USP18
IRF9
GFAP
IFIH1
IFI35
DDX58
IFIT1
CBR4
TAS1R2
UGT8
GPR119
TSHR
HRH3
COMT
QRFPR
ARX
KCNQ4
CHRM5
GIPR
KRTAP26−1
PCDHA8
EEF2
GAPDH
SLC46A1
PTH2R
SLC19A3
SIX3
SLC27A2
RLN3
GZMB
PCDHGB5
CD14
Astrocyte
Oligodendrocyte
OPC
Sst
Vip
SG
CD48
PABPC1
CD28
NOP53
EEF1A1
CTCF
CCR7
CD2
RSL24D1
EIF3H
NACA
FBL
SLC5A6
LAT
SLC27A5
WNT3A
EOMES
SLC7A9
CD8A
TBX21
IFNG
GZMH
SLC27A2
GPR55
ZAP70
SLC22A3
PDCD1
STAT4
CD86
TNF
SLC52A3
SLC46A1
APOE
SLC6A14
CD14
SLC12A8
ERBB2
CD80
KCNQ4
DMD
TP63
SOX2
T CD4
T CD8
Macrophage
LNG
HUWE1
CD28
CD2
CD4
TMSB10
RAF1
GZMB
CD8A
STAT4
CD48
IFNG
HNRNPA1
ANXA2
CDC42
ISG15
CD86
CD14
FPR1
TLR4
CD209
S100A8
S100A9
CCR2
CSF3
TLR5
C1QA
TLR2
TREM1
CYBB
RSAD2
C1QB
CD80
TREM2
CXCL11
COL4A2
CCL20
APOE
CXCL5
T cell
Monocyte
Macrophage
