# Supplementary material for: HCNetlas: A reference database of human cell type-specific gene networks to aid disease genetic analyses
Source: PLoS Biol. 2025 Feb 5;23(2):e3002702. doi: 10.1371/journal.pbio.3002702 (PMC11798474; doi:10.1371/journal.pbio.3002702)
Supplement: Attachment — Submitted filename: PLoS_response_letter_final.docx [file pbio.3002702.s014.docx]

**Responses to referees (our responses in bold or colored text)**

**Manuscript ID: PBIOLOGY-D-24-01455R1**

**Attached please find our revised manuscript. We do appreciate the reviewers’ critical but valuable comments, which have been a guideline in revising the manuscript. We have provided revised manuscript, Figure 2C, Figure 3A, Supplementary Figure S4. We believe that the revisions improved the manuscript particularly in delivering our analysis to the readers clearly. We hope that our revised manuscript is suitable for the publication.**

**= = = = = = = = =**

Reviewer #1:

Summary of the paper:

The authors introduced HCNetlas (human cell network atlas), a collection of reference cell type-specific gene networks (CGNs) from a wide array of healthy tissue cells, and used them as the reference network to compare with disease CGNs. For the comparisons of disease vs healthy CGNs, they developed three network analysis methods, differential compactness, differential hubness, and differential pathways, to investigate cell type-specific functions of disease genes. They applied their methods to systemic lupus erythematosus (SLE), Alzheimer's, and lung cancer disease tissue scRNA-seq samples to identify disease-associated cell types, genes, and pathways.

The novelty of this paper lies in the development of reference CGNs and their application to disease data to reveal the cell types most affected by diseases. This could facilitate the development of cell-type-resolved diagnostics and therapeutic strategies for complex human diseases. Overall, the paper is original and of good quality, but needs to address the following questions.

Major Comments:

Q1: An individual's disease network consists of both disease-related gene and network alterations and the individual's biological/genetic differences. Using multiple individual samples may not necessarily eliminate all the biological bias. The paper mentioned 1000 single cells as the cutoff for constructing the CGNS. It is not clear whether the paper used multiple samples to construct the CGNs (both healthy and disease). How many samples are needed to produce a robust network? Some discussion on this will be helpful.

We appreciate the reviewer’s question. While we anticipated an effect of cell count on network construction, we did not specifically consider the impact of sample number. This is because we have applied the same network inference method for constructing personal CGNs, which rely on data from a single sample. Additionally, other groups have successfully constructed single-cell gene networks for individuals in various studies. Therefore, we intentionally developed a network inference method that can work with single-cell transcriptome data from even a single individual.

Nevertheless, we tested the effect of sample number on network construction in a manner similar to cell count analysis: by examining whether there is a correlation between sample number and network size across CGNs. As expected, we did not observe a significant correlation between donor number (x-axis) and network size, as measured by edge/node count (y-axis), though there was a slight increase from 1 donor to 2 donors (see Figure below).

Although the sample number does not affect the efficiency of network inference, it may influence the properties of the model. A network derived from a single sample reflects the personal characteristics of that cell type, whereas a network constructed from many samples will capture the core properties of the given cell type. We have added a discussion on the effect of sample number on CGNs in the revised manuscript (line 573).

***“Moreover, healthy control samples may vary in properties such as gender, age, body mass index, smoking status, diet, and other lifestyle factors. The effects of these variables will be mitigated by including cells from a large pool of healthy donors, leaving only the core gene network that represent the given cell type within the range of a healthy state.”***

Q2: In evaluating the cell-type-specificity of CGNs (Method), the healthy CGNs are constructed using scHumanNet, a program developed in their previous paper. Please give some basic introduction to the scHumanNet method so readers do not have to go back to read the previous paper.

We thank the reviewer for this comment as this increases readability. We have added the following text in the methods section (line 94):

***“Briefly, scHumanNet takes a single-cell transcriptome count matrix for a specific cell type, followed by data imputation, transformation and normalization. It then produces a transformed gene activity score matrix and filters the reference interactome, HumanNet, for gene pairs in the matrix that pass a minimum activity score, creating a CGN. The edge weight scores are inherited from the reference interactome.”***

Q3: (Method) They tested the correctness of CGNs by assuming that genes functionally connected within a CGN reflect the properties of their respective cell type (defined by the GO annotation). They considered the interconnectivity within genes for each cell type as a measure of the cell-type specificity. This is true, but how can this prove the correctness of edges (or each edge) in CGNs?

We constructed CGNs using a reference interactome, HumanNet. This approach to CGN inference assumes the high accuracy of the reference interactome and select reference network edges that are likely to exist in the given cell type. As a result, the correctness of the CGN edges is predetermined by the reference interactome. Notably, the accuracy of HumanNet edges has been shown to outperform other popular reference human interactomes, as demonstrated in a previous study (*Nucleic Acids Research 50: D632, 2022*).

They identified hub genes within each CGN and ranked them using the FindAllHub() function of the scHumanNet. Again, how to prove the correctness of these hub genes?

We apologize for insufficient description of the *FindAllHub()* function. This function randomly shuffles the constructed network and creates a null distribution of centrality values. This provides statistical significance for the hubness of a gene of interest. We have added this description in the revised manuscript as follows (line 121).

***“FindAllHub() function randomly shuffles the constructed network and creates a null distribution of centrality values. This enables the calculation of statistical significance for the hubness of a gene of interest.”***

Q4: (Results) HCNetlas. Figure 1C has many tissue abbreviations. Explanations of these tissues should be displayed somewhere (moved from the supplementary tables to the main text).

As suggested, we provide an additional table for tissue abbreviation as Table 1 in the main text.

Why does the CGN network size reach the plateau when the cell count reaches 1000? Why does the network size shrink when the cell count goes over 1000?

The network inference algorithm tests the expression of gene pairs that interact in the reference interactome within a given cell type using single-cell transcriptome data. If both genes pass the likelihood threshold, their interaction is included in the CGN. Single-cell transcriptome data is often sparse, with many dropout events. This sparsity can be mitigated by pooling cells in the gene expression analysis. The number of pooled expression values is determined by the total number of cells. Consequently, when fewer cells are available, the likelihood of both genes passing the expression threshold decreases, potentially missing cell-type-specific edges. As the number of cells increases, more edges meet the paired expression criteria, leading to a larger network size. When the dataset reaches around 1,000 cells, the network size reaches its maximum range for the given cell type.

However, we are uncertain whether network size decreases when cell count exceeds 1,000, as there are limited networks generated from more than 1,000 cells, resulting in a wide range of error model, as shown in Supplementary Figure 1A.

The network gene expression profiles of the same cell types tend to cluster together in Figure 1B. Aren't the cell types defined by gene expression profiles? Figure 1B can only confirm that the program chose a similar set of genes for the same cell types, but can't prove whether these genes are related to the cell type or not. Can you explain these?

The given network profile is based on connected genes rather than expressed genes. Because connections of each gene depend on not only its expression but also expression of other genes for the cell type’s identity and function.

Cell-type-specificity of individual links for the CGNs were evaluated separately by using DBs for cell type specific genes (Fig. 2A-C).

Q5: (Results) HCNetlas as a tool for unraveling cell type specificity of disease genes. They ranked genes in each CNG by network degree centrality and then conducted gene set enrichment analysis using disease-associated genes obtained from DisGeNET and GWAS. For Figure 3A, how are the disease gene sets (rows) ordered? Are they ordered randomly? How are the CGNs (columns) ordered hierarchically? What do the colors/numbers represent? The conclusions drawn from Figure 3A are confusing.

We thank the reviewer for pointing this out. Both row and column were not ordered in any specific manner and is the result from hierarchical clustering with default parameter. The default parameter of R package ComplexHeatmap cluster.rows and cluster.cols were set to TRUE. The heatmap color values represent scaled ssGSEA enrichment scores.

To make this clearer, we have added the following text in the Methods section as follows (line 133):

***“Hierarchical clustering of disease gene sets and CGNs was performed using the R package ComplexHeatmap, with the default parameters, where cluster.rows and cluster.cols were both set to TRUE.”***

We have revised Figure 3A as follows with labels for color intensity (scaled ssGSEA score):

We also revised the Figure 3A. legend as follows (line 780):

***“Each column represents a CGN of HCNetlas, while each row corresponds to a disease gene set sourced from either DisGeNET or GWAS Catalog, ordered by hierarchical clustering. Color intensity indicates scaled ssGSEA enrichment values for the disease gene sets (rows), reflecting the degree of association of the CGN signature genes with each disease terms.”***

Q6: (Results) What are the definitions of gain and loss of pathways? A pathway consists of multiple genes. How to compute them?

To define the gain and loss of pathways, we introduce a simple metric called *diffQ* as described in Methods section of the original manuscript. This metric compares the ratio of significance of pathway overlap between the reference CGN and the disease CGN. For a specific pathway, we test its overlap in the two groups of CGNs, with a lower p-value indicating a stronger association with the group. According to the formula, a positive *diffQ* value signifies that the pathway has greater overlap in the disease group compared to the reference group (gain of pathway), while a negative value indicates the opposite (loss of pathway).

We apologize for any confusion caused by the organization of the methods section. The description regarding the gain or loss of pathways belongs to the section 'Cell type-resolved genetic analysis of Alzheimer’s disease (AD) with HCNetflas' in the original manuscript. To enhance clarity, we reorganized the methods and added a new subheading at line 193.

***“Evaluation of differentially associated pathways between reference and disease CGNs***

Considering the association of gain of hubness and lost of hubness genes with AD in inhibitory and excitatory neurons, …”

Q7: (Results) How many disease samples were used in the SLE, AD, and Lung Cancer data analysis?

As described in the original manuscript (line 143), In the SLE, we acquired scRNA-seq data for peripheral blood mononuclear cells (PBMCs) from 41 patients with SLE and 15 healthy controls as reported by Nehar-Belaid *et al.*[1]. We excluded two SLE patient samples lacking SLEDAI scores to ensure a consistent analysis. Therefore 39 disease samples were analyzed.

We apologize for not providing sample number information for AD and lung cancer case studies in the original manuscript. Regarding AD data, single-nucleus RNA sequencing data from 12 patients were acquired from Morabito *et al.* [2]. For lung cancer CGN construction, we used scRNA-seq data from 29 tumor tissues provided by Qian *et al*. [3]. We have added this information (the number of AD patients and the number of lung cancer samples) in the revised manuscript (line 183 and line 211, respectively).

Q8: I found a related paper in the following: Li, Z., Liu, G., Yang, X. et al. An atlas of cell-type-specific interactome networks across 44 human tumor types. Genome Med 16, 30 (2024). <https://doi.org/10.1186/s13073-024-01303-w>

We thank the reviewer for pointing out this article and giving us the opportunity to highlight our work’s difference compared to CellNetdb proposed by Li et al. CellNetdb focuses on providing resources for cell-type-specific network in the context of a specific disease, cancer. They have extensively collected 44 tumor types and constructed CGNs with the SCINET algorithm, using various interactomes as the reference including HumanNet. CellNetdb provides additional information such as enriched pathway terms, somatic mutations, and TCGA survival results based on the modeled network nodes.

We want to emphasize that hcNETLAS consists of CGNs from healthy individuals, which can serve as reference CGNs for comparison with disease CGNs to investigate cell-type-specific characteristics of various diseases. This is particularly advantageous, as many single-cell transcriptome studies, especially in cancer, often lack suitable control sets. Unlike expression-based studies, where comparing disease data with healthy control data from different datasets requires caution due to batch effects, network-based studies are relatively robust against such effects. We provided a proof-of-concept study demonstrating that leveraging network topology can help overcome the challenge of batch effects. By utilizing reference CGNs from healthy controls, we propose a framework to potentially map the 'gene-cell-disease axis' using single-cell expression data from disease tissues.

Although there were not many 'reference' datasets available during our stages of data collection, we were able to effectively demonstrate through multiple examples—SLE, AD, and lung cancer datasets—that this approach can be leveraged to capture meaningful results.

We have added the following text (line 543) in the discussion section of the revised manuscript to illustrate this point.

***“Notably, while our work was under review, CellNetdb [4], a database of CGNs across 44 tumor types, was published. Although its CGN inference strategy is similar, CellNetdb is specifically designed to investigate cell-type-specific cellular and molecular mechanisms focused on cancer. In contrast, HCNetlas serves as a database of reference CGNs for studying a wide variety of human diseases.”***

Reviewer #2:

In their manuscript Yu et al describe the setting of a new resource database for the collection of cell type-specific gene networks (CGNs) within specific cell types of various tissues. They want to establish reference CGNs, and then for any given set of disease genes be able to compare to the reference set of CGNs. Thus, the idea behind their project is to make available for researchers a resource of control CGNs that could be used for disease-related gene analysis instead of using matched control samples. Overall, the paper is well-written and include validation with the use of specific disease for assessing differences in CGNs.

Although the idea behind the study is interesting and valuable, I am unsure if at this stage with its application to few datasets, if HCNetlas brings enough for investigators to use it as their controls and what are the plans for extension. Why did the authors limit their CGNs to a few datasets, instead of applying it to all control datasets available in the HCA? It seems that for now HCNetlas is limited to data from 2 published papers, comprising in total 25 organs and the associated 61 cell types identified in these 2 reports. One of these papers is mostly limited to immune cell identification, and thus limiting the whole human being aspect of the analyses and CGNs identified. At this stage, it seems a better fit for any immune-related disorder as most of the cell types included for CGNs are immune cells.

We appreciate the reviewer’s constructive comment. When we began this project in early 2022, there were not many 'control' datasets available at the atlas level (please refer to the HCA data portal at Projects - HCA Data Explorer (humancellatlas.org)). Additionally, it was challenging to manually verify whether individual studies adequately reported underlying health conditions of the samples to confidently include them as references. Therefore, we selected three (not two) published atlas datasets at that time: (1) the Tabula Sapiens data, (2) the Allen Brain Atlas, and (3) the Conde et al. immune cell atlas dataset, which was part of the Human Cell Atlas project. We trusted that the authors of these datasets would have made every effort to collect samples suitable for use as references.

Please note that we have already mentioned this concern in the discussion section of the original manuscript as follows (line 568):

***“Consequently, our endeavor to create compendium of true reference CGNs was limited by the availability of atlas level resources with varying health conditions (not necessarily diseased). However, this limitation is expected to diminish as the field of single-cell transcriptomics continues to grow. As more data are generated, particularly for healthy tissues, it will become feasible to construct a more comprehensive array of CGNs, covering a wider variety of cell types.”***

HCNetlas will continue to expand as reference datasets grow. During the manuscript review, we recognized the need to update our references due to the significant accumulation of HCA reference data, and this expansion will be carried out in the near future.

Unless I am misunderstanding, I am unsure why the authors limited their approach to 2 datasets, albeit 2 very large datasets. If the goal is to eliminate running control samples, this approach would need to be widened and seems limited for now as a general database. It is well-known and described by several sc studies performed on human subjects that the donor-donor variability is huge. So, the limitation here seems to reduce the catalog of 'control samples' to a limited number of donors, 28 which may definitely not be enough to cover all ages for both sexes. Is the goal to extend to all available control datasets deposited in the HCA? This may need to be discussed and added as limitations.

We apologize for any lack of clarity on this matter. The three (not two) large datasets we used to create reference CGNs were carefully analyzed for their suitability in disease research. Our intention was not to present the initial work on HCNetlas as a comprehensive database resource, but rather to emphasize its analytical framework, which we effectively validated across various disease categories—an autoimmune disorder (SLE), a neurological disorder (AD), and a cancer (lung cancer)—as proof of concept.

We agree with the reviewer’s concern regarding donor-donor variability, which is indeed a major challenge in studies using clinical samples. The primary goal of this work is to present an analytical framework for using CGNs generated from cell atlas data to dissect gene-cell type-disease relationships. We acknowledge the limitations of our reference CGNs due to the limited number of donors in the cell atlas. However, as cell atlas projects continue to grow and incorporate more donors, the inclusion of cells from a larger pool will help mitigate donor-associated variability in the network models.

We have added the following text to discuss this concern (line573):

***“Moreover, healthy control samples may vary in properties such as gender, age, body mass index, smoking state, diet, and other lifestyle factors. The effects from these variables will be mitigated by including cells from a large pool of healthy donors, leaving only the core gene network that represent the given cell type within the range of a healthy state.”***

At different parts in the paper, the authors mention using the studies from Dominguez Conde 2022 and the Tabula Sapiens from 2022, as well as the Allen Brain atlas data. However, in the text, they mention using HCA data. Could it be clarified for readers?

We apologize for any confusion. As suggested, we have clarified the datasets used in the revised manuscript as follows. All other instances of 'HCA' refer to the project itself rather than the datasets used in our study.

***(line 241) “To build reference CGNs, we utilized scRNA-seq data from immune cell atlas project [5] and The Tabula Sapiens [6], as well as single-nucleus RNA-sequencing (snRNA-seq) data from the Allen Brain Atlas [7].”***

***(line248) “To investigate this aspect, we conducted an analysis of CGNs derived from various cell atlas datasets,…”***

A minimum of 1,000 cells for a given cell type for CGN identification may remove from analysis some important cell type that may be related to diseases. This is a major limitation of the study.

We agree that this is currently a major limitation for investigating rare cell types in a disease context. However, we believe that by incorporating additional datasets in future work, this challenge will be addressed. To improve clarity, we have added the following text in the revised manuscript (line 588).

***“To avoid this issue, we constructed CGNs only from datasets containing at least 1,000 cells. However, this approach may exclude some important cell types from the database.”***

On a more philosophical point, there are different factors that could alter gene expression and thus CGNs in human cell types that would not be disease-related but may affect disease-related genes. Or they may be specific conditions that may later CGNs without causing a specific disease. Every human is unique, and may have unique sets of CGNs at distinct point in time, that may vary with aging, although not all of these people will present with a specific disease. How is this taken into account in HCNetlas? What are considered controls from non-controls? Do you plan on applying specific threshold for inclusion in your database, like age, BMI, smoker or not, etc…. Healthy for one cell type may not be healthy for another, and the way control tissues are handled at biobank is limited in terms of metadata. For example, obtaining a healthy tissue, may just mean that this tissue did not present with a tumor, but not that the person in which this tissue was derived was healthy, and thus the CGN may be affected. With such variability how can a reference atlas be handled?

We agree that including genuinely healthy control samples is a critical issue when collecting reference CGNs for HCNetlas. In fact, the Human Cell Atlas (HCA) Consortium faces the same challenge, as their goal is to collect and provide single-cell omics data from healthy tissues to serve as references for human cell biology. Therefore, it is reasonable to expect that the HCA team puts significant effort into ensuring the quality of reference samples for their work. If we are provided with detailed metadata, additional data filtration could be considered. However, in principle, we may rely on the sample annotations provided by the HCA team, which should be acceptable given that both HCA and HCNetlas aim to provide reference cellular models (gene expression and gene networks, respectively).

Regarding the potential effects of various sample properties that might be present in the healthy samples, combining cells from a large number of samples may help mitigate these effects, leaving only the core gene-gene interactions for the given cell type within the range of a healthy state.

We have added the following text to discuss this concern (line572):

***“Moreover, healthy control samples may vary in properties such as gender, age, body mass index, smoking state, diet, and other lifestyle factors. The effects from these variables will be mitigated by including cells from a large pool of healthy donors, leaving only the core gene network that represent the given cell type within the range of a healthy state”***

Reviewer #3:

The authors created and tested co-expression based gene-gene networks for human immune and brain cells. They used these networks to identify disruption of specific cell types based on patient samples from Alzheimer's disease and systemic lupus erythematosus.

Given the word 'atlas' in the title, I assumed this approach would highlight specific cell types body-wide for a given set of disease-associated genes. Instead, this is limited to immune cells from several organs and the brain cell types (which didn't cover the resident immune cells). In this regard, readers might need clarification on how comprehensive this network atlas is.

We appreciate the reviewer’s comments. The name HCNetlas was coined based on our endeavor to create cell type specific networks based on single cell atlas datasets and ultimately leverage these CGNs as a framework for disease research. We indeed show in this manuscript that this analytical framework is feasible. We have collected all available atlas-level single cell references at the time of launching this study, and we have plan to extend the reference atlases in the near future as adding more CGNs for comprehensiveness is now possible due to expansion of the HCA projects.

To improve clarity, we have revised the discussion section (line 526):

***“…making use of HCNetlas—an analytical framework for leveraging compendium of reference CGNs derived from a single-cell expression atlas of healthy individuals for disease research.”***

GCNs created from the control samples from the SLE and Alzheimer's disease datasets were not compared to results from the HCNetlas GCNs. This needs to be tested since the authors claim their approach can 'circumvent the need for matched control samples.' This may highlight that the AD study took samples from the prefrontal cortex while the HCNetlas GCN is derived from the motor cortex, and was also enriched for neurons. This was done for the lung cancer study, but the conclusion that 'lung cancer genes exert their roles in immune cells' doesn't seem supported, given that the HCNetlas GCN was only from immune cells.

We appreciate the reviewer’s question. To test the compatibility between the CGNs from HCNetlas (HCA) and those generated from healthy samples in the dataset (HD), we examined the overlap of network nodes and found a high degree of overlap for the same cell types (Figure below, left). Additionally, we analyzed the overlap of statistically significant differential hubs (Q-value < 0.05, determined using *FindDiffHub()*) and observed a high overlap coefficient between HCA and HD across cell types (Figure below, right). These results support the compatibility of CGNs generated from healthy samples in the dataset with those provided by HCNetlas.

We also evaluated the prediction rate of SLE-associated genes based on hubness in the CGN for myeloid cells, the SLE-associated cell type. As illustrated in Fig. 4C, the disease myeloid CGN inferred from disease samples demonstrated a higher prediction rate (below left, AUROC = 0.789) for SLE-associated genes compared to the reference myeloid CGN from HCNetlas (below center, AUROC = 0.650). Interestingly, the myeloid CGN inferred from healthy samples (HD) exhibited a lower prediction rate than disease myeloid CGN but similar to that of the reference myeloid CGN from HCNetlas (below right, AUROC = 0.690), further emphasizing the alignment between healthy control CGNs from HCNetlas and those inferred from healthy samples in the dataset.

We were unable to perform a similar test for AD due to the lack of available healthy samples in the dataset used for the study. This situation, in fact, underscores the usefulness of HCNetlas, which can provide healthy control CGNs for comparison with disease CGNs.

Regarding the reviewer’s comment that 'the conclusion that lung cancer genes exert their roles in immune cells doesn’t seem supported, given that the HCNetlas GCN was only derived from immune cells,' we would like to clarify that we are not concluding immune cells are the sole cell type associated with lung cancer. We acknowledge the limitation of our current study, as most of the cell types examined are immune and neuron cells. However, we observed that many genes with high hubness, reflecting their functional significance within the system, are well-known lung cancer genes (as shown in Fig. 6a and c, proportional bar plot, orange). For validation, we gathered these lung cancer genes from widely-used databases such as Cancer Gene Census, CancerMine, and IntOGene. These databases catalog genes that, when mutated or non-functional, are causally implicated in cancer, making them a highly credible source of lung cancer-associated genes. With validation from independent sources, our results suggest that many lung cancer genes may play roles in immune cells. Nevertheless, we agree that we should soften this claim in the manuscript and have revised the text accordingly (line 26).

***“Additionally, they suggest that many lung cancer-related genes may exert their roles in immune cells.”***

Figure 2 suggests that the GCNs are partially driven by expression level, given the high connectivity of the B and T cell marker genes. That leaves the question of whether the 'mere changes in expression levels' would highlight the same cell types.

We infer network links based on the likelihood of expression between gene pairs in the reference interactome. Thus, 'correlated changes' in the expression of two genes connected by the reference interactome, rather than changes in individual genes, provide insights into the same cell types.

For Alzheimer's disease, the GWAS-associated genes are expressed highly in microglia cells in the brain, which were not included in HCNetlas. The authors did not find this because they lacked the microglial cells to generate an GCN. In addition, the Alzheimer's GWAS hits are enriched for expression in the spleen and macrophages. This links the peripheral immune system to the disease. These associations were found from body-wide atlases like GTEx, but it doesn't seem HCNetlas would highlight macrophages. So, given its current setup, it would have missed this important characterization of the Alzheimer's disease genes.

We appreciate the reviewer’s insightful comment. We were not aware of association between macrophage and Alzheimer’s Disease, and did not assess its connectivity in the macrophage/myeloid compartments. We assessed the normalized compactness using the immune reference cells used for HCNetlas. Indeed, as the reviewer anticipated, we found high connectivity of AD GWAS hits (585 genes, collected from all terms with “Alzheimer” included in the term name of the GWAS catalog) within the macrophage and myeloid CGNs.

We have added this new result as **Supplementary Figure S4C**, and the following text in the revised manuscript (line 337):

**“****Additionally, AD-associated genes from the GWAS were found to be highly connected within the macrophage and myeloid CGNs (Supplementary Figure S4C), consistent with the association between AD risk and macrophage transcriptional network [43].”**

Methods

-were the author-provided cell type annotations used? It seems that they were not and CellTypist was used instead. This should be clearly stated and justified, as the author-provided annotations should be trusted over CellTypist predictions. In addition, the CellTypist parameters should be specified.

For the Conde et al. dataset, the authors have provided the curated form of CellTypist result as the cell type label, and we have directly used this annotation. We also agree with the reviewer that author provided annotation should be used. The original authors did not specify the parameters run, but explicitly wrote that the manual curation process involved: 1) examination of expression of known marker genes and marker genes derived from CellTypist models; 2) cross-validation and cross-prediction with CellTypist training dataset and two independent datasets from the human gut and lung.

The method section was revised as follows (line 85):

***“We used the author-provided cell type labels, which were initially annotated by CellTypist and subsequently underwent manual curation.”***

Minor

-for Figure 2C, please mark which genes are within the top15 of the specific four cell types. It seems there is some overlap since only 50 genes are shown.

We have revised figure 2C, highlighting genes in bold that are only found in only in one cell type, and revised the manuscript legend of figure 2 as follows (line 776):

*“****Genes uniquely found in each cell type are highlighted in bold****”*

-Figure 2 uses GO BP, but KEGG is used later on. GO is more frequently updated and comprehensive and should be used over KEGG.

We acknowledge that GO terms are frequently updated and are more representative of cellular functions. However, in the context of disease research, GOBP is a suboptimal database for testing gene set enrichments, as the top candidate gene sets often highlight essential housekeeping functions, regardless of the disease context (as they rightfully should). For this reason, we specifically chose to use the KEGG database. Although it may not be updated as frequently, the terms in KEGG often represent disease-relevant functions, such as 'Pathways of neurodegeneration,' 'Pathways in cancer,' and 'Prion disease.'

-the source of the disease-associated gene sets is highly variable. DisGeNET and KEGG collects genes from many sources with different degrees of evidence. This is clear with the AD genes as 17% are NADH:Ubiquinone Oxidoreductases, which lack genetic evidence and probably end up in the list because KEGG links them together.

We appreciate the reviewer’s insightful comment. We did not manually curate the disease gene sets but rather prioritized the comprehensiveness of the gene sets (with varying but existing evidence). For the AD-related genes, we removed NDUF-associated genes and re-analyzed their overlap with the constructed CGNs. As shown below (left), the conclusions drawn from the initial gene set (right) remain consistent, with only minor changes in the hypergeometric test p-values.

**********

**Thank you again for your time and effort, and for assisting in improving the manuscript. We look forward to your positive response.**

**References**

1.	Nehar-Belaid, D., et al., *Mapping systemic lupus erythematosus heterogeneity at the single-cell level.* Nat Immunol, 2020. **21**(9): p. 1094-1106.

2.	Morabito, S., et al., *Single-nucleus chromatin accessibility and transcriptomic characterization of Alzheimer's disease.* Nat Genet, 2021. **53**(8): p. 1143-1155.

3.	Qian, J., et al., *A pan-cancer blueprint of the heterogeneous tumor microenvironment revealed by single-cell profiling.* Cell Res, 2020. **30**(9): p. 745-762.

4.	Li, Z., et al., *An atlas of cell-type-specific interactome networks across 44 human tumor types.* Genome Med, 2024. **16**(1): p. 30.

5.	Dominguez Conde, C., et al., *Cross-tissue immune cell analysis reveals tissue-specific features in humans.* Science, 2022. **376**(6594): p. eabl5197.

6.	Tabula Sapiens, C., et al., *The Tabula Sapiens: A multiple-organ, single-cell transcriptomic atlas of humans.* Science, 2022. **376**(6594): p. eabl4896.

7.	Sunkin, S.M., et al., *Allen Brain Atlas: an integrated spatio-temporal portal for exploring the central nervous system.* Nucleic Acids Res, 2013. **41**(Database issue): p. D996-D1008.
